# Balancing creativity and pragmatism: insights from a curriculum for interprofessional learning at Linköping University

**DOI:** 10.5116/ijme.6931.a9c2

**Published:** 2025-12-19

**Authors:** Maria Kvarnström, Susanne Kvarnström, Elin A. Karlsson

**Affiliations:** 1 Department of Health, Medicine, and Caring Sciences, Linköping University, Linköping, Sweden

**Keywords:** Interprofessional education, interprofessional learning, curriculum design, health professions education, medical education

## Abstract

**Objectives:**

This study explores the application of the current curriculum, launched in 2016, for interprofessional learning (IPL) at the Faculty of Medicine and Health Sciences at Linköping University, Sweden.

**Methods:**

Perceptions from students, teachers and key persons (n=19) were investigated with focus groups and individual interviews. The interviews explored perceptions of curriculum design, implementation, and interprofessional learning activities. A four-dimensional framework for curriculum development and evaluation constituted the theoretical lens for the analysis. Specifically, a qualitative directed content analysis was used.

**Results:**

Four overarching categories were identified. 1) “Aiming towards high quality healthcare professionals working together naturally”, included the arguments for IPL within healthcare and why the curriculum revision was conducted. 2) “Comprehending how to work in a group, a team, and an interprofessional team”, related to the desired learning outcomes and competencies. 3) “All on board? – The search for meaningful learning activities for students and teachers that the programs embrace”, reflected on how learning activities were designed and experienced. 4) “Enhancing legitimacy and the provision of organizational prerequisites for the curriculum”, described the organisational structures for enhancing the legitimacy of the IPL curriculum, as well as the role of teachers.

**Conclusions:**

This study emphasizes balancing pedagogical innovation with pragmatic considerations for successful curriculum application. The findings highlight the need for experienced teachers, organisational support, and meaningful learning activities that align with both program-specific and interprofessional outcomes. Despite challenges, the current curriculum at Linköping University shows potential as a model for integrating interprofessional learning into health professions education.

## Introduction

The need for future healthcare to rely on interprofessional collaboration is emphasised globally as being essential to meet the demands placed on health care systems.^1^^, ^^2^ Scandinavian countries mirror the global challenges with an aging population and healthcare staffing being critical issues.^3^ For the health care professions to achieve the required interprofessional competencies, health professions educations (HPEs) around the world rely on including interprofessional education (IPE) in order for students to learn with, from and about each other to improve collaboration and the quality of care and services (as the definition of IPE^4^^, ^^5^). IPE can be organised in different ways (design of the learning activities/module and whether it is mandatory etc.) and with different degrees of magnitude, such as a fully integrated curriculum for interprofessional learning (IPL) at faculty level as an umbrella, or other approaches with shorter modules and various numbers of educational programmes participating for each module. There are abundant examples of curricula for IPL in the literature e.g., ^6^^, ^^7^ which include options for developing IPL curriculum. Studies on how a comprehensive IPL curriculum is revised, implemented and applied, looking into the curriculum as a whole are however scarce, and previous research on IPL more often focuses on students learning outcomes and factors that may facilitate, or hinder, implementation.^8^^, ^^9^

Linköping University, Sweden, has a strong tradition of IPL, implementing one of the first systematic curriculum for IPL in 1986 at a European medical faculty.^10^^,^^11^ Further, the other cornerstone - as a pedagogical approach alongside IPL at the medical faculty - is problem-based learning (PBL) which has also featured since 1986. The combination of IPL and PBL may enrich the learning process, for instance by bringing about social stability as well as mutual respect among future healthcare professionals.^12^ An interprofessional training ward (IPTW) was implemented in 1996 at Linköping University Hospital’s Department of Orthopaedics. This was the first interprofessional training ward for undergraduate students in the world.^13^ In 2012, the changing context, like the health care system and institutional pre-requisites regarding both teachers and students, resulted in a project at the faculty to revise the existing IPL curriculum at Linköping University.^14^

The curriculum prior to the 2016 revision consisted of three modules, encompassing a total of 12 weeks of full-time studies (18 higher education credits, HECs) with different themes as described by Wilhelmsson in 2009.^10^ Later (in 2011) the theme of the second module was changed from sexology to improvement science in a clinical setting. The changes in the revised curriculum included a design to promote integration during the first module by delivering it side-by-side with program-specific content and designing PBL scenarios that would align the IPL module with the program course (e.g., a scenario with the same patient, focusing on different aspects). The current curriculum no longer includes the general introduction of PBL, since the first IPL module has been postponed to mid-term and the introduction of PBL at the beginning of the first semester has now been allocated to the individual programs. Further, the interprofessional training wards were expanded to include interprofessional training units at primary healthcare centers. The duration of the entire curriculum was reduced from twelve weeks to eight weeks (12 HECs).

In the current curriculum, the students work together on general topics such as health theory and ethics in the first module, while the second module includes a project on improvement science in practice. The different professional roles are further highlighted and intertwined in the third module which is conducted on student-driven interprofessional training wards and interprofessional training units at primary healthcare centers, supervised by clinical tutors. The participating undergraduate programs are biomedical laboratory science (BMLS), medicine (M), nursing (N), occupational therapy (OT), physiotherapy (PT), and speech and language pathology (SLP), although not all programmes participate in all modules or necessarily during the same semester. This is further described in a previous study.^15^ In 2021, all programmes participated in all modules.

The intended learning outcomes for the students are related to the Core Competencies for Interprofessional Collaborative Practice (IPEC) which include four domains: 1) Values and ethics, 2) Roles and responsibility, 3) Communication, and 4) Teams and teamwork,^16^  as well as a fifth domain identified at Linköping University: Learning and pedagogical processes.^14^ The modules are described in more detail by Lindh Falk and colleagues.^17^ Alongside the activities in the three IPL modules, there are also other interprofessional learning activities involving students from two or more different programmes, such as interprofessional simulations (for instance a sequential simulation with a stroke scenario and seminars).

This study is a continuation of our previous study, which explore the intentions and outcomes of the revised curriculum via a qualitative study including documents (syllabuses, study guides, educational program plans and supervisor guides) and complementary interviews with key persons.^15^ In the previous study, we found, a reduction in the variations between programmes in terms of how the IPL modules are articulated after the revision. The findings also pointed towards the importance of an IPL coordinator as a supporting organizational factor, and high expectations on teachers to enact the intended curriculum.^15^ Important lessons when revising an existing fully integrated IPL curriculum in health professions education programs were identified, such as the importance of creating learning activities that are feasible and comprehensible for a broad group of teachers and students, which needs to be further explored from the perspectives of different actors to get a better understanding of the  application of the current curriculum. As the findings from the previous study were based on documents and complementary interviews with key persons, we wanted to deepen the knowledge of aspects emerging from the documents, through interviews. The current study explores the perceptions of the application of curriculum from students’, teachers’ and key persons’ perspectives, contributing new knowledge. In this context, the term application refers to the application of curriculum put into practice. Given that the Faculty of Medicine at Linköping University has maintained and developed an extensive curriculum for IPL since 1986, with mandatory participation for all students, the current study holds significant interest for a broad spectrum of educators. The long-standing tradition and robust structure warrant investigation and dissemination of findings in an international context, potentially serving as a model and inspiration for a fully integrated IPL curriculum. Further, the current study will respond to the need for studies on how a curriculum as a whole is revised, implemented and applied.

The aim of the study was to explore the application of the current curriculum for interprofessional learning at Linköping university.

The research questions were: 1) How is the application of the current curriculum in IPL perceived by different actors? and 2) Which prerequisites exist in order to apply the current curriculum?

## Methods

This was a qualitative interview study, in which we used a theory-based evaluation in accordance with Lilliedahl and colleagues.^18^ and the framework for curriculum development and evaluation by Lee and colleagues^19^: the four-dimensional curriculum framework (4DF). The latter entails four interrelated dimensions for curriculum development processes: 1) The future orientation of health practices; 2) The desired competencies and capabilities for the students; 3) Activities for teaching, learning and assessment; and 4) Organizational requirements, supporting institutional delivery,^19^ which we used as a structured tool to interpret and theorize the empirical findings.

### Data collection

Data were collected using a purposive sampling strategy,^20^ which is suitable for obtaining a broad range of experiences from different stakeholders’ perspectives. In this study, the stakeholders where key persons involved in the development of the current curriculum, teachers, and students. For all stakeholders, interview guides were developed inspired by findings from the previous document study,^15^ as well as questions connected with the four dimensions of the theoretical framework.^19^

Four key persons were invited to participate in separate interviews, all of whom agreed to do so. All interviews were performed by SK and conducted during May 2023. The interviews were carried out either at the participants’ work premises or in their home and lasted between 60 and 90 minutes. In addition to background data, the semi-structured interview guide for key persons consisted of each respondent’s perceptions of the process of designing and implementing the revised curriculum, as well as specific questions that had arisen in connection with the preceding document study in the research project.^15^ For instance, reflections on why a specific learning activity, the cut-out PBL scenarios, included in the redesigned curriculum did not turn out as intended as well as reflections on the suggested mentoring system for teachers.

Teachers who had recently been involved in one or more learning activities in the curriculum were selected to ensure variation among educational programs, gender, and experience as a teacher in higher education and in interprofessional learning activities. The teachers had experience of the interprofessional modules, with roles such as facilitators for PBL tutorial groups and seminar leaders. In this study, we refer to all of them as teachers. The teachers were divided into two groups. One group included the teachers with significant experience from both the current and previous curriculum (teachers, group 1), while the other group included those who had been involved in the IPL modules for one to two years (teachers, group 2). In total, 17 teachers were invited to participate in a focus group interview, of whom 11 participated in the interview. The primary reasons for not participating were a lack of time or not being available at the time of the interview. The focus group interviews with teachers were conducted at Linköping University in May and October 2023, by MK and EK, and lasted for approximately one hour each. The semi-structured interview guide for teachers included questions about their prerequisites to apply the IPL curriculum including if anything is missing regarding the prerequisites and about the role as teachers and support to fulfil the task. Questions about their experiences of the learning activities were also included, for instance about the connection between the three modules and how the modules in the revised curriculum works compared to the previous curriculum (the latter question was for the first group only, with teachers who had been involved in both the current and the previous version).

For the students, we approached all those who had participated in at least two (preferably all three) of the IPL modules and who were currently studying the latter parts of their respective programs on any of the six educational programmes at Linköping University. However, we received few responses. A few students contacted the researchers to ask whether they would be paid to participate but this was not the case for this study. Due to the limited responses and the difficulties recruiting students, we used various channels to spread the information and the invitation to participate in this study. We e-mailed students directly based on lists of participants from the second and third IPL modules. Several reminders were sent. We provided all six programme directors for the respective educational programs with the information letter and asked them to pass this on to all students on the last semester of their program, including asking them to send reminders. Social media was used, whereby we asked students to share the information letter in groups on, for instance, Facebook. Lastly, we chose to extend our recruitment strategy to also include snowball sampling by encouraging those who had agreed to participate in the study to ask fellow students who they thought might be interested in participating. We also asked students’ associations to share the information letter. In total, over three hundred students were approached, of whom four ultimately participated in focus group interviews (students, group 1 and 2 respectively). The students agreed to participate by filling in a digital form, and we thus do not know which of the above recruitment strategies were most successful. However, the majority of the participating students agreed to participate at a stage when only e-mails (either from us or from their programme directors) were used. The focus group interviews with students were conducted by MK and EK through a video meeting in January 2024 and lasted about 55 minutes each. The students were asked, for instance, about their experiences of cooperation with students from other programs, their prerequisites for adopting the curriculum and learning about IPL, how they perceive that the IPL modules are connected with each other, and the support from the teachers as facilitators for the IPL.

In total, four key persons, 11 teachers, and four students participated in the interviews.

### Participants

By ‘key persons’, we refer to people who held positions with a mandate at a significant executive level, and who were involved in the developmental process for the revised IPL curriculum. All the participating key persons had clinical backgrounds as health care professionals.

The participating teachers had a broad range of experiences from various fields, including physiotherapy, occupational therapy, medicine, nursing and economics. In total, they had experience from all existing IPL modules, but primarily the first two, for instance, as examiners, lecturers and PBL group tutors. In the more experienced group (teachers, group 1), the mean age was 56 and in the group of newer teachers (teachers, group 2) it was 41 years. The more experienced teachers had a mean of 14 years of experiences as teachers in IPL and the corresponding length of experience for the newer teachers was two years, although both groups had additional experience as teachers in higher education. Six of the teachers were female while five were male.

The students represented three educational programs: nursing, occupational therapy and medicine. Two were male and two were female and they were between 24 and 28 years old.

The Swedish Ethical Review Authority concluded that ethical approval for this study was not needed (Dnr 2022-06875-01). This study was conducted in accordance with the ethical principles of the Declaration of Helsinki.^21^ For instance, all participants were provided with written information about the study and were informed that they could withdraw their participation at any time. This information was distributed to them in connection to the invitation to participate. In order to ensure confidentiality of the key persons, they were allowed to read the manuscript before submission and give their approval. Further, to prevent them from being identifiable the descriptive information about the key persons in the methods section needed to be somewhat general.

### Data analysis

The interviews were recorded and transcribed verbatim and were analysed with content analysis using a directed approach^22^ where the four dimensions of Lee and colleagues (4DF)^19^ constituted the theoretical framework. Preliminary categories were then developed. This analysis included both manifest and latent features, and a process including skimming, careful reading and interpretations of patterns within the data. This iterative process requires focused re-reading and review of the coding and category construction.^22^

In this study, transcripts were initially skimmed, before being read more carefully as meaning units were discovered and categorised. The first author performed these steps using the software NVIVO and discussed potential codes and categorisation with the other authors. Four main categories were created in accordance with the chosen theoretical framework by Lee and colleagues.^19^ Initially, all authors read a selected part of the transcripts and discussed potential coding and categorization in order to calibrate and to ensure a purposeful analysis. The analysis was thereafter performed by the first author and continuously discussed with the other authors. Subcategories were then developed, quotations were selected, and the results were written. The use of NVIVO facilitated a transparent process in which all authors were involved in the analysis to some extent. The analysis and preliminary findings were also discussed and validated during a seminar with colleagues in medical pedagogics.

## Results

The analysis resulted in four main categories in accordance with the 4DF by Lee and colleagues.^19^ The first category “Aiming towards high quality healthcare professionals working together naturally”, included the arguments for IPL within healthcare and why the curriculum revision was conducted. The second category, “Comprehending how to work in a group, a team, and an interprofessional team”, related to the desired learning outcomes and competencies for the IPL modules. The third category, “All on board? – The search for meaningful learning activities for students and teachers that the programs embrace”, reflected on how learning activities were designed and experienced by the actors. This category included four subcategories: 1) Designing meaningful interprofessional learning activities, 2) Problem based learning as pedagogical tool, 3) Integrating IPL into the respective programs, and 4) Pragmatic pedagogics. Lastly, the fourth main category, “Enhancing legitimacy and the provision of organizational prerequisites for the curriculum”, described the organisational structures for enhancing the legitimacy of the IPL curriculum, as well as the role of teachers. This category entailed three subcategories: 1) Promoting legitimacy and a common ground through organisation, 2) Structure and progression of the interprofessional modules in the curriculum, and 3) The role, support, and prerequisites of teachers.

### Aiming towards high quality healthcare professionals working together naturally

The first category includes the intention of shaping students into high quality healthcare professionals with good prerequisites to work in teams, in order to align the IPL curriculum with overall health practice orientations. The intentions of conducting a revision were also highlighted in terms of wanting to update the curriculum in accordance with national and international standards and needs, as illustrated by the below quotation from one of the key persons.

“The Lancet report and the global conversation also meant that we had to look at our local situation from that perspective. Are we on track? Are we meeting what is expected at an international level? Or are we just looking at our own needs?” (Key person 4)

The benefit of actually meeting students from other programs, whom students might eventually need to work with, is mentioned by both students and teachers. There is also a positive attitude towards including interprofessional activities early on in the education which was highlighted by teachers as stated below. 

“I think it's important that they [the students] meet early on, and there is research that shows exactly this interprofessional value. That we will actually work together, and you haven’t created a lot, carry a lot of preconceptions with you as well. But that you also kind of start to see what is in common. Because that’s the idea of the first semester as well. What is common for us who are going to work in healthcare?” (Teachers, group 1)

### Comprehending how to work in a group, a team, and an interprofessional team

In this category, competences and knowledge that are needed to work in interprofessional teams and how those are developed during the interprofessional curriculum are presented. This is exemplified below by a student who describes how, during the IPTW, they gained a wider understanding of what they decided to include in the documentation, what could be important for the other professions and thus should be included. Further, it also reveals knowledge and competences that were developed but might not be related to interprofessional learning and collaboration.

“It also clarifies the simple things I can do in my profession to make life a lot easier for other people. But it will be... You may sometimes understand why you do things. Why is it important that this is included in this documentation? Yes, but that’s because this is interesting to this person. It’s highly uninteresting to me. But it’s very good for someone else. So, it will be a way to show care, I think.”  (Students, group 1)

“It has become a case of memorising of a lot of laws and other things here and there that you [the students] don’t even understand and can hardly apply.” (Teachers, group 1)

Distinctions between learning about working in a group, teamwork in general, and interprofessional teams are highlighted. For instance, students describe how they have learned to work in groups, and state that some of the modules facilitate learning about how to work in a group with other individuals, rather than actual interprofessional collaboration. Key persons describe how the IPEC domains were integrated into learning outcomes and how there was an intention of allowing the students to do and discover IPL - rather than simply talking about IPL – even though theories of IPL are not a prominent part of the content of the modules in the curriculum.

“People think that just because you are in a team, you are being interprofessional. And I don’t think you are. But there has to be an awareness of, like, this is the patient's problem. This is what I can contribute. In what way do we need to adapt what you contribute so that this turns out in the best possible way for the patient? In other words, this negotiation must take place in a conscious way. Not just that the medical parts come first and then the rest.” (Key person 4)

### All on board? – The search for meaningful learning activities for students and teachers that the programs embrace

The third main category relates to how the interprofessional learning activities are perceived to be designed and enacted, the usage of PBL as a tool in the learning activities, and the challenge of integrating the IPL modules into the respective programs. Further, the results encompass the negotiation between being pragmatic and creative when it comes to pedagogics, and that the design of learning activities must be achievable and accepted by a broad range of teachers and students.

### Designing meaningful interprofessional learning activities

The teachers emphasise the importance of creating meaningful IPL activities that spur curiosity and new experiences. However, they also state that some of the learning activities tend to focus on formalities, such as how to write a report and how to use references properly and that their students seem to focus on what will be examined, rather than considering the lifelong learning for becoming good healthcare professionals. In line with this, the students describe a lack of meaningfulness in relation to some of the examination forms, as described below.

“And I think that the administrative work that is involved in, for example, creating a poster or creating a report or just creating some common document, that’s not what needs to be examined. But what should be examined is what kind of knowledge do we have, that we have acquired specifically by working together?” (Students, group 1)

The students emphasize that the time available and informal discussions influenced their possibility to learn and to spur the curiosity of their peers. Commonly the third IPL module (clinical wards) is highlighted as meaningful, and the second (improvement science) as too hectic although they appreciated the collaboration with real healthcare units.

“Both during... IPS stroke and IPTW, there was time to talk about other things in a completely different way. There was a lot of watercooler-talk. People sat and chit-chatted with each other between patients or in the lunchroom. You talked about things that had nothing to do with the profession and it also meant that, I don’t know, you created an understanding of the person and in turn, they became a lot more inviting, and you were more curious about what they had to offer.” (Students, group 1)

Further, key persons describe that students were not satisfied with the second module including improvement science. Previously the topic had been sexology which according to the key persons and the experienced teacher group was more appreciated among the students. However, to change the topic to stroke simulation as suggested by the persons responsible for the revision, was not carried out due to, for instance, objections from healthcare representatives.

### Problem based learning as pedagogical tool

The experienced teachers highlight the fact that PBL is now introduced by the respective programs (and is no longer included in the first IPL module). Teachers, students, and key persons all state that the notion of PBL, and in particular tutorial groups, can mean different things as the programs tend to use it in various ways. The students describe how the knowledge of PBL and being in tutorial groups helped them feel comfortable and get started with tasks, although they acknowledge the variations between programs as illustrated by the following quotation.

“So even though all the programs have worked to some extent with tutorial groups, this work is very differently designed in the different programs, the way I have understood it anyway. Even though the basic idea is that it should be the same, I have certainly not got the feeling that it is. I think that could affect when you put together a group and then you have to have a tutorial group. Because everyone has a slightly different view of what a tutorial group is and what it is expected to do.” (Students, group 2)

Although students perceive these variations as problematic for their cooperation with students from other programs, key persons acknowledge that variations are a natural feature of PBL:

“I think it has turned out well because it forced the programs to also take ownership, and you can think differently, I mean all programs have their kind of PBL, a little, but I think you have to let them do that too, because ownership also includes being able to do a little bit as you want.” (Key person 3)

### Integrating IPL into the respective programs

The findings demonstrate a challenge regarding how to integrate the IPL modules properly into the respective programs and their content. In particular, the first module is highlighted by the participants, in terms of spending half the time on IPL and half the time on the respective program. The initial idea of cut-outs, with the aim of facilitating an integration between program-specific content and IPL, has been abandoned although the half-and-half design remains. Specifically, the more experienced teachers highlight this and state that this becomes problematic for the students who work with two completely different things in parallel, but they cannot see a connection:

“But you’ve lost this back and forth with the program… I feel like I kind of miss that. Because now I don’t think the point remains with them having half weeks in the same way, and the students don’t understand that either. I can’t really defend that, because we don’t have the integration anymore.” (Teachers, group 1)

There are variations in the prerequisites provided to the students. For instance, students highlight that the medical- and nursing program was more hectic compared to the other programs, and although they were supposed to devote half their time to IPL in the first module, the students perceived that the program-specific content did not decrease when the IPL module started, which made it difficult for them to participate on equal terms.

“My feeling is that it has been a little different between the programs, that not all programs have had a break in the main program, so to speak, during these [IPL] weeks. But during the IPTW there may have been a greater break in these [program- specific] activities. But otherwise there have been assignments or lectures or other things that need to be done on your main program, with you having to solve another task for a whole new group at the same time. And that, in turn, I think may have interfered a bit with it, because... yes, but the motivation to put all the focus on IPL is not quite as much then when you have something else that you need to do at the same time.” (Students, group 1)

Further, the students highlighted that students from some programs were better prepared for the IPL modules, depending on the content during the rest of the semester on their respective program. This is also highlighted by key persons, in terms of how the subject itself on the respective programmes can facilitate or hinder the conditions for mixing different areas of knowledge and being able to integrate these. For instance, it may be easier to create a clear connection between IPL and ethics or professionalism in healthcare compared to cell biology.

### Pragmatic pedagogics

This subcategory relates to the negotiation between creating pedagogical and creative learning activities, and relating this to pragmatic features such as time, budget and other resources. For instance, in a diverse group of teachers it is essential that everyone is onboard (students, teachers and programs) and able to deliver meaningful learning activities to students. One example of a learning activity that was perceived as too complex for teachers to assess and to be retained in the curriculum was the use of portfolios. Key persons describe that this faded away, as it was not properly implemented and no one took responsibility for it. In general, key persons highlight the difficulty of anchoring the curriculum so that students, teachers and programs agree on how the curriculum is to be enacted.

“But then it’s about sustainability when it gets rolling. Staff, people and thoughts are being replaced. It starts to twist in the corners as you wonder what is going on here. It’s another quandary if you think that you have a responsibility to keep this alive. To find sustainability in this without becoming rigid. Because I think it's a challenge to be creative but also to keep a tight rein.” (Key person 1)

Further, the reduction in time allocated to the IPL curriculum and the budget, as directed by the faculty, made a pragmatic approach even more necessary.

“All programs are a zero-sum game with weeks, and everyone wants more. So that's it, you have to use the weeks you have wisely. But there has been criticism for a long time from various quarters that it (IPL) has taken too much time, and those voices got the faculty’s ear and then they [the faculty] put their foot down and decided that this is how it should be.” (Key person 3)

Besides the key persons, this is also reflected in the interviews with teachers. Both teachers and students describe that there is a lot to do in a short amount of time in the IPL modules, in particular relating to the first two modules. The tasks and learning activities risk being managed as a checklist and hampering students’ learning:

“They try to squeeze in so much material that you don’t have time to reflect independently. Instead, there are always [teacher-]led activities, and they usually always point towards what the final reflection “should” be.” (Students, group 1)

### Enhancing legitimacy and the provision of organisational prerequisites for the curriculum

The fourth main category include the organisational structures for enhancing the legitimacy of the IPL curriculum, in terms of a common ground at faculty level and the structure and the progression of the modules in the curriculum. Further, the role of the teachers and their prerequisites including support are part of this main category.

### Promoting legitimacy and a common ground through organization

The first subcategory relates to how organisational aspects and strategies influence the process of designing and delivering the interprofessional modules. Furthermore, the balance between the content and design of the profession specific part of the educational program and the interprofessional modules is emphasised as important by both teachers and key persons. Support from the head of the faculty and organizational prerequisites, e.g., a program director for the interprofessional curriculum, are described as essential.

### Structure and progression of the interprofessional modules in the curriculum

This subcategory includes the choices when designing and revising the curriculum, and the subsequent effects on the students’ opportunities for interprofessional learning. Further the findings relate to the challenges of designing for a progression and to what extent there is a progression. Both students and teachers discussed whether the IPL modules should be early or late in the educational programs and that there are pros and cons with the different choices. It seems difficult to see the progression to some extent, while on the other hand the idea behind it is also perceived as evident and clear as described by one of the key persons:

“It’s the three-step rocket that’s the thing, and I mean there is so much focus on IPL 1 [the first module], but the idea is that IPL 1, to my mind, it's kind of the starting point and then you do one, it’s like the triple jump, and then you make a starting point in improvement knowledge and it could have been something else, but I actually think it’s pretty neat as well and it’s out in clinical practice. I still think our curriculum is a flagship, I think it’s great. And then, like, you have to run a ward together and put this in and then like, the students are so inexperienced, they can’t run a ward, but they still have to form that team, and they do it fantastically well, it’s a miracle, every interprofessional placement is a miracle in itself... Then maybe people aren’t so whiny, that’s my projection kind of, but I think it turned out great. I’m super proud of what we introduced.” (Key person 3)

### The role, support and prerequisites of teachers

This subcategory relates to the teachers and includes several aspects. For instance, the recruitment of teachers and the assignment of the task at the faculty are discussed, and several teachers express the need for experienced teachers, in line with the recommendations for the IPL modules. But at the same time teachers state that it is not uncommon for novice teachers to be assigned to the interprofessional modules, which might be challenging. This sends a signal about the lack of significance that these modules are given, which is exemplified by a quotation from one of the newer teachers:

“I’m wondering if the demands are so high, if you can bring in someone who has never been involved in PBL and who comes from a completely different discipline, and just ”Yes, now you’re going to start supervising this”. If there is any control on such requirements really… It will be a strange signal if it says in the documents that there are high demands. If it is to be experienced teachers. I don’t know anything about it, but let’s do this!” (Teachers, group 2)

The support for teachers in terms of teacher guides is described in partly contradictory ways. This highlights the importance of clear guidance, referred to as being even more important since there is a large group of teachers involved and a rotation between the semesters as well as novice teachers. On the other hand teachers also want the opportunity to use their pedagogical experience and skills without being too controlled or burdened with administration.

“It's a perspective that our employer actually misses. How are we treated as teachers in this? Are we treated with respect? No, not at all. And then comes one of those papers. How are we going to live up to that when we haven't been given the conditions to do so? We want to, I think all of us, every single day we want to learn new things. And we do that by being a PBL facilitator. But then we have to work in a problem-based way. Absolutely. Not getting a long list of checkpoints. What do I become then? I don't even have to be there. You can tick these off when you've done this stuff. And then there, you are deprived of your... your profession.” (Teacher, group 1)

## Discussion

This study aimed to explore the application of the current curriculum for interprofessional learning, specifically focusing on the perceptions of different actors and the prerequisites to apply it. The findings demonstrate how creative pedagogical ideas had to take a backseat as there were challenges in getting the heterogenous group of teachers and students on board when delivering the IPL-curriculum. Pragmatic aspects adopted included reducing or revising learning activities and making these less complex. Further, the integration between program-specific content and interprofessional content seemed to vary between programs and between the three IPL modules. The expressed need for experienced teachers within the IPL modules was not adhered to, leading to unexperienced teachers constantly being assigned to IPL-modules, sending a clear signal that the curriculum is not important. This is also related to poor prerequisites for teachers and the lack of opportunities to design creative learning activities that are feasible to deliver. Students describe that what they have been doing in the IPL modules tends to be more basic groupwork than actual interprofessional teamwork, in contrast to the curriculum’s intention revision of shaping students into high quality healthcare professionals with good prerequisites to work in interprofessional teams. Keeping in pace with the surrounding world is essential for educating health care professionals equipped with the current and future health care landscape. The role of IPE will remain essential as the workforce changes and technology is continuously advancing.^23^ One way to facilitate the link between education and practice, and to stay updated, is to use the IPEC-domains^24^ and the updates on these. This study reveals that the IPEC domains were integrated into learning objectives for the IPL modules, but that the students do not work on theories of IPL and what this involves. This study highlights the value of actually meeting students from other programs and learning together, which both teachers and students describe. However, the students describe how they have learned about groupwork, rather than actual interprofessional teamwork. This raises the question of what interprofessional education and interprofessional teamwork actually are, and how to make the students discern interprofessional dimensions of teamwork and achieve interprofessional collaborative competencies. CAIPE defines interprofessional education as the “occasions when two or more professions learn with, from and about each other to improve collaboration and the quality of care”.^5^ The importance of possibilities for informal interprofessional learning described by the students is in line with other studies.^25^

Both teachers and students in this study highlight the importance of meaningful learning activities that spur curiosity. The approaches to learning and the approaches to teaching are thus essential. For instance, examination forms for the interprofessional modules need to be perceived as relevant for both students and teachers, and not as being too focused on formalities rather than interprofessional competencies. In this study, students expressed that they did not fully understand why they were examined in, for instance, writing a report as an IPL learning activity. The students found this less meaningful or more unclear in terms of identifying the interprofessional aspects rather than simply conducting academic writing with others. The students took a positive view of learning with each other but seemed to find it difficult to discern the learning from and about each other in some of the learning activities. They also expressed that there were too many activities to carry out, meaning that they did not have time for reflection. A useful concept relating to this, may be the concept of constructive alignment,^26^ that relates approaches to teaching with approaches to learning. In order to increase the opportunities for learning among students and to enable a heterogeneous student group to meet the course objectives, teachers need to plan the design of learning activities carefully.^26^^,^^27^ Possible consequences of not integrating the knowledge of students’ learning with the design of teaching and the desired goal fulfilment could be that only those students who are already high-performing will succeed in passing the exam, or that the class in general will be dissatisfied with the course if the learning activities are not designed in line with set course objectives. The results of our study indicate that there is a risk that the balance between the content and pedagogical design of the learning activities and assessments on the one hand and administrative and formal aspects on the other hand could be tipped towards the administrative and formal aspects, resulting in more time and effort being spent on e.g., finalising a formal report rather than learning together about the content of the report.

The balance between pedagogical design and pragmatism is also an important factor regarding the realisation of the curriculum and the included modules, with the intended integration by delivering the first IPL module alongside program-specific courses as an example. The pedagogical design of integrating the PBL scenarios in both program-specific and interprofessional terms did not remain as intended, and subsequently the half-and-half -IPL versus program-specific content is no longer as clearly understood and meaningful. The findings indicate some challenges when it comes to organising the curriculum, with recruiting and supporting PBL facilitators being a crucial aspect. The findings from the present study are in line with previous findings^15^ when it comes to intentions not being fulfilled, as there appear to be novice teachers in the facilitator team. The importance of trained teachers to facilitate the students’ learning process is described in recent reviews^7^^,^^28^ further supporting the pivotal role of teachers and highlighting a need to examen aspects relating to training and recruiting teachers for interprofessional learning in greater depth. Further, in terms of providing organisational support, the appointment of a program director for IPL is an example of the crucial support at faculty level, an issue that is further discussed by Clark who e.g., conclude that IPE is vulnerable to shifting goals of higher education.^29^ The role of a program director might provide legitimacy and a common ground, as discovered in this study, which in the long run potentially could facilitate stability through winds of change within the faculty. The necessity of collaboration across the faculty is mentioned in a recent review,^28^ as the problem of predominately siloed structures - as well as an already content-heavy curricula – can hinder the development of a curriculum for IPL.

### Strengths and limitations

This study was conducted through the lens of a theoretical framework suitable although not solely used within the field of medical pedagogics. The use of 4DF as the framework for curriculum development has been described by others, for instance in relation to pain medicine, physiotherapy and for postgraduates.^30^^-^^32^ The publication describing the framework in 2013 ^19^ has been cited more than 60 times and the 4DF is now an established framework for curriculum development.^33^ In this study, the 4DF was used as an overarching lens guiding the interview guide, analysis, and presentation of findings. Through the deductive analysis, aspects of interest that did not fit the theoretical framework might have been lost. However, theory provides not only structure but also new perspectives of the findings, which was useful in this study. Potentially also other more specific theories could have been used. However, multiple theories and a deductive approach entail a risk that aspects of interest are being sidelined.

The study’s trustworthiness will be discussed below in terms of transferability, credibility, dependability and confirmability.^34^ A strength of this study is the representation of several different stakeholders, increasing confirmability through the triangulation of data. The number of students participating was, however, low, which is a weakness in this study. The representation of different stakeholders might also have a positive impact on the transferability of the findings, as different perspectives are represented. The use of theory might also facilitate transferability although this study only represents one single setting (i.e., the medical faculty at Linköping university). However, the purpose of this study was not to establish generalizable findings but rather to provide an example of how curriculum revision can be carried out and perceived, ultimately serving as inspiration to others. Although Linköping University has had an interprofessional curriculum for quite some time, there are eminently challenges and a need for continuous development. Few studies explore the “how” of curriculum development, and this study contributes with such an example. Future research could preferably focus more on conducting comparative studies using data from various settings. Patton^34^ describes credibility as using rigorous methods while paying attention to the researcher’s experience, and philosophical beliefs. The design and analysis of this study has been discussed extensively both within the research group and also with other researchers through, for instance, seminars. To some extent this might also have had a positive impact on the dependability as the work on the study was kept transparent and a structured research process was facilitated through discussions with others.

Trustworthiness can also be related to the concept of information power, in which high quality research can be discussed in relation to the precision of data retrieved, the experience of researchers, as well as the use of theory.^35^ Malterud and colleagues^35^ argues that recruiting participants relevant for the study, possessing rich information relating to the study's aim, would reduce the need for an extensive sample. In this study, we recruited participants with unique insight into the curriculum from different perspectives. For the carefully selected key persons, all who were invited to participate indeed participated. The final sample of teachers represented several educational programs and different experiences as teachers in higher education. The group of teachers was discussed in the research group and assessed as satisfactory. Although the students were few, they possessed experiences that they expressed clearly and nuanced in the focus group interviews, sometimes also relating this to what they had heard from their peers. We would argue that this study holds power in the depth of information retrieved, although we acknowledge that we would have preferred additional students, which was attempted through various recruitment strategies.

Regarding reflexivity, the data was analysed according to Braun and Clarke,^36^ and all three researchers involved have experience in teaching and research within the field of interprofessional learning. However, they did not engage in the organizational aspects of curriculum re-design and delivery or serve as module directors but were partially involved as teachers. Reflexivity was fostered through continuous and constructive reflection on potential biases throughout the research process. We recognize that preconceived notions can hinder strict analysis; therefore, maintaining a meta-perspective on our assumptions and their influence on our analysis was a recurring theme in the collaborative research process.

### Implications

This work provides insights into the research field of comprehensive curricula for IPL, especially the revision, implementation and application. The lessons learned from Linköping University may be valuable as inspiration for other universities across the world.

Future studies could focus more on the organisational aspects, such as the prerequisites for teachers, and the impact this might have on the design of learning activities. Further, future research could investigate how to create meaningful, well integrated learning activities that generate possibilities for the students to develop the required interprofessional competences. Such studies could broaden the picture by using other methods. The authors of this paper are currently working on a survey study that builds upon the findings from this study and a previous study^15^ to further explore teachers ‘and student´s understandings of the IPL curriculum.

## Conclusion

The development and application of a revised IPL curriculum is a complex and resource-intensive endeavor. This study highlights the importance of balancing pedagogical innovation with pragmatic considerations to ensure the successful enactment of the curriculum. The findings underscore the need for experienced teachers, organisational support, and meaningful learning activities that align with both program-specific and interprofessional learning outcomes. Despite the challenges, the revised curriculum at Linköping University demonstrates the potential to serve as a model for integrating interprofessional learning into health professions education.

### Acknowledgements

We would like to thank the participants for contributing their knowledge and experience.

### Conflict of Interest

The authors declare that they have no conflicts of interest.
